# Non-Invasive Imaging of Vascular Inflammation

**DOI:** 10.3389/fimmu.2014.00399

**Published:** 2014-08-18

**Authors:** Enrico Ammirati, Francesco Moroni, Patrizia Pedrotti, Isabella Scotti, Marco Magnoni, Enrica P. Bozzolo, Ornella E. Rimoldi, Paolo G. Camici

**Affiliations:** ^1^Cardiothoracic Department, San Raffaele Scientific Institute and University, Milan, Italy; ^2^Cardiovascular and Thoracic Department, AO Ospedale Niguarda Ca’ Granda, Milan, Italy; ^3^Unit of Medicine and Clinical Immunology, Department of Medicine, San Raffaele Scientific Institute and University, Milan, Italy; ^4^CNR Istituto di Bioimmagini e Fisiologia Molecolare, Segrate, Milan, Italy

**Keywords:** vasculitis, non-invasive imaging, contrast-enhanced ultrasound, vascular inflammation, positron emission tomography, cardiovascular magnetic resonance

## Abstract

In large-vessel vasculitides, inflammatory infiltrates may cause thickening of the involved arterial vessel wall leading to progressive stenosis and occlusion. Dilatation, aneurysm formation, and thrombosis may also ensue. Activated macrophages and T lymphocytes are fundamental elements in vascular inflammation. The amount and density of the inflammatory infiltrate is directly linked to local disease activity. Additionally, patients with autoimmune disorders have an increased cardiovascular (CV) risk compared with age-matched healthy individuals as a consequence of accelerated atherosclerosis. Molecular imaging techniques targeting activated macrophages, neovascularization, or increased cellular metabolic activity can represent effective means of non-invasive detection of vascular inflammation. In the present review, novel non-invasive imaging tools that have been successfully tested in humans will be presented. These include contrast-enhanced ultrasonography, which allows detection of neovessels within the wall of inflamed arteries; contrast-enhanced CV magnetic resonance that can detect increased thickness of the arterial wall, usually associated with edema, or mural enhancement using T2 and post-contrast T1-weighted sequences, respectively; and positron emission tomography associated with radio-tracers such as [^18^F]-fluorodeoxyglucose and the new [^11^C]-PK11195 in combination with computed tomography angiography to detect activated macrophages within the vessel wall. Imaging techniques are useful in the diagnostic work-up of large- and medium-vessel vasculitides, to monitor disease activity and the response to treatments. Finally, molecular imaging targets can provide new clues about the pathogenesis and evolution of immune-mediated disorders involving arterial vessels.

## Introduction

Vasculitides are conditions defined by the presence of inflammation of the vessel wall, with progressive alteration of the lumen, including lumen stenosis, occlusion, or even aneurysmal dilation. They can be broadly divided into infectious vasculitides, characterized by direct invasion of pathogens in the vessel wall and non-infectious vasculitides. The latter, also known as primary vasculitides, encompass a heterogeneous group of immune-mediated disorders, classified, according to the size of vessels that are predominantly involved, into small-, medium-, and large-vessel vasculitides (LVV) (1). The diagnosis of small and medium vessels vasculitis is generally based on clinical findings, serological markers, and histological evaluation (2). LVV include giant cell arteritis (GCA), Takayasu arteritis (TAK), primary central nervous system vasculitis (PCNSV), and chronic periaortitis (CP) (3). The most common LVVs are GCA and TAK in which the aorta and its main branches are generally involved. GCA occurs more frequently in older adults, showing a predilection for the temporal artery and other extracranial vessels (4). Clinical manifestation of this disease ranges from ischemic symptoms and signs (such as jaws claudication) to aneurysmal rupture (4, 5). TAK is more prevalent in adolescent girls and young women, with a strong predilection for the aortic arch and its branches, in particular the subclavian arteries (up to 98%) and common carotid, although also pulmonary and coronary arteries (<10%) may be involved. The involvement of the major branches of the aorta is much more marked at their origin than distally, leading to clinical manifestations that ranges from arm claudication to myocardial infarction. Different classification criteria for LVV have been defined, and all of them are based upon clinical presentation, evidence of inflammation, and vascular abnormalities (6–8). However, they have proven largely unsatisfactory for diagnostic purposes (9, 10), frequently leading to delayed diagnosis (11). Even once the diagnosis is established, accurate monitoring of disease activity and response to therapy is not an easy task (5). Extensive clinical evaluation of the arterial tree is currently recommended (12), and imaging plays a major role in this setting. *In vivo* detection of inflammation within affected blood vessels may provide a reliable tool to assess disease activity, leading to better clinical management of the patient. Although imaging techniques are particularly useful to diagnose and monitor LVV, they can also play a role in the work-up of medium-vessel vasculitides, classically represented by polyarteritis nodosa (PAN) in adults and Kawasaki disease (KD) in children (13, 14). In contrast, current imaging techniques are unable to visualize small vessels, thus we will focus on the role of imaging studies in diagnosing and monitoring LVV, including some applications in medium-vessel vasculitides and autoimmune systemic disorders with potential vascular involvement such as Behcet’s disease.

Inflammation of the vascular wall is characterized by different pathological changes, such as edema, vasa vasorum activation and proliferation (15, 16), alteration of endothelial homeostatic function, and immune cells infiltration (4, 17, 18), ultimately leading to anatomical remodeling with consequent functional alteration. Knowledge of the biological basis of these processes has led to the development of imaging strategies aimed at identifying them *in vivo*, mainly with the development of probes directed to key molecular targets (19). Imaging techniques allowing for molecular imaging include ultrasonography, positron emission tomography (PET), most often associated with computed tomography (CT), and magnetic resonance imaging (MRI), all of which will be discussed in this review. Table 1 summarizes the main features of the imaging techniques used to study patients with vasculitides. Furthermore, inflammation of arterial vessels is a fundamental pathogenetic element in atherosclerosis and associated clinical manifestations. Compelling evidence of the link between atherogenesis and inflammation has built over the last decades, leading to the current hypothesis that atherosclerosis is not merely a disease due to passive lipid accumulation in the vascular wall but an active, immune-driven process (17, 18). Not surprisingly, many diseases associated with systemic inflammation due to immune alterations are associated with increased risk of cardiovascular (CV) morbidity and mortality due to atherosclerosis (20, 21) that cannot be fully explained by traditional CV risk factors, suggesting a role for immune activation (22). Therefore, characterization of inflammation within atherosclerotic plaques by means of molecular imaging may identify patients at risk for disease progression or acute clinical manifestations (23, 24).

**Table 1:** **Features of main non-invasive imaging technique for vascular imaging**.

Imaging technique	Form of energy	Spatial resolution (mm)	Availability
**Ultrasonography**	High frequency sound waves	0.1-1	Widespread
**CT**	X rays	0.3-1	Widespread
**MRI**	Radiofrequency waves	0.2	Large centers
**PET**	Photons annihilation	4-6	Large centers

*PET: positron emission tomography; CT: computed tomography; MRI: magnetic resonance imaging*.

## Ultrasound

Ultrasound imaging is widely available, inexpensive, and repeatable and does not involve the use of ionizing radiation. It is generally performed by an experienced sonographer using high-quality Doppler ultrasound equipment and linear probes > 8 MHz (25, 26). Examination typically comprises B-mode ultrasonography, which depicts anatomy using a gray scale, and Duplex ultrasound, which combines color Doppler ultrasound and pulsed Doppler ultrasound to display information about blood flow and to estimate blood flow velocities (26). The main limitation of ultrasound imaging is that it cannot depict structures below bone or air. For this reason, it does not provide reliable information about the thoracic aorta, unless performed via a transesophageal approach. In addition, acquisition of ultrasound images is operator dependent, although studies of vascular ultrasound have shown high rates of interoperator agreement (27, 28). In the following sections, we will review the main ultrasonographic findings in blood vessel inflammation, which are summarized in Tables 2 and 3.

**Table 2:** **Summary of main ultrasonographic findings in inflamed blood vessel, together with their pathological correlate and clinical significance**.

**Sign**	**Alteration**	**Pathological correlate**	**Clinical significance**	**Vasculitis**	**Reference**
**Halo sign or Macaroni sign**	Hypoechoic concentric thickening of blood vessel wall	Edema	High sensitivity and specificity for LVV diagnosis; potential role for follow-up	GCA (halo sign) and TAK (macaroni sign)	(29–32, 38–40)
**Increased common carotid artery intima-media thickness (IMT)**	Thickening of common carotid artery vessel wall	Vascular remodeling under pathological stimuli	Increased risk for CV events	All conditions associated with high CV risk, including vasculitis	(41–43)
**Stenosis, occlusion or aneurysmal dilation**	Reduction (stenosis and occlusion) or increase in vessel caliber; flow alterations	Advanced pathological remodeling of blood vessels	Cause of ischemic symptoms and signs; risk of aneurysmal rupture; patient follow-up	All	(3, 5)
**Adventitial neovessels with contrast-enhanced ultrasound (CEUS)**	Moving bright spots on the adventitial layer of the vessel wall after microbubbles administration	Neoangiogenesis due to inflammation*	May correlate with vasculitis activity	TAK and GCA	(48–50)

*Correlation with neovessel formation has been demonstrated for atherosclerosis. Pathological correlation studies for vasculitides have not yet been performed.

**Table 3:** **Summarizes key studies concerned with imaging of inflammation in blood vessels in LVV, and their main results**.

Technique	Study	Number of patients	Results	Reference
**Color Doppler ultrasound**	Ball et al., The British Journal of Surgery, 2010	998	Meta-analysis of 17 studies showing a sensitivity of 69% and a specificity of 89% for the halo sign in temporal artery	(32)
	Arida et al., BMC Muscoloskeletal Disorders, 2010	504	Meta-analysis of 8 studies showing a sensitivity of 68% and a specificity of 91% for the halo sign in temporal artery	(31)
	Maeda et al., Ultrasound Med Mol, 1991	23	“Macaroni” sign detected carotid artery involvement in 19 out of 23 patients with TAK	(37)
	Habib et al., Clin Rheumatol, 2012	32	Halo sign decreases after a mean of 21 days from beginning of therapy	(39)
**Contrast-enhanced ultrasound**	Schinkel et al., European Heart Journal Cardiovascular Imaging, 2013	7	Ultrasonographic contrast allowed better delineation of carotid arteries lesions. It also allowed vessel wall neovascularization in five out of seven patients (TAK or GCA)	(50)
**Transesophageal ultrasound**	Bezerra Lira-Filho et al., Journal of the American Society of Echocardiography, 2006	14	71% of thoracic aorta segments were found to be thickened, and 37% dilated in the 14 TAK patients studied by transesophageal echocardiography	(60)
	Espinola-Zavaleta et al., Echocardiography (Mount Kisco, NY). 2005	15	In the studied TAK patient cohort, 67% of patients had aortic regurgitation, 60% mitral or tricuspid regurgitation and 33% reduced coronary reserve measured with contrast enhancement	(61)
**CT angiography**	Khandelwal et al., European Journal of Radiology, 2011	15	CT angiography showed variable thickening of aorta and main branches in patients with active TAK	(65)
	Prieto-Gonzalèz et al., Annals of the Rheumatic Diseases, 2012	40	CT angiography was able to detect large-vessel involvement in 67% of patients with GCA. The proportion was higher for treatment naïve patients (77% vs 29%)	(73)
	Kang et al., Radiology, 2014	111	53% of patients had coronary artery involvement, while only 29% were symptomatic for heart disease	(72)
**PET using FDG**	Besson et al., European Journal of Nuclear Medicine and Molecular Imaging, 2011	101	Meta-analysis of six studies on patients with GCA, showing a sensitivity of 80% and a specificity of 89% for FDG-PET	(81)
	Blockmans et al., Arthritis and Rheumatism, 2006	35	Vascular FDG uptake was shown in 83% of 35 patients with GCA. It decreased after 3 months of effective therapy, but no further decrease was documented at 6 months follow-up	(88)
	Fuchs et al., European Journal of Nuclear Medicine and Molecular Imaging, 2012	30	PET was shown to increase diagnostic accuracy for LVV from 54 to 71%	(87)
**PET with PK11195**	Pugliese et al., Journal of the American College of Cardiology, 2010	15	PET/CT allowed visualization of tracer uptake in the vessels of all the six patients with active disease, but in none of the controls	(96)
**Magnetic Resonance Imaging**	Mavrogeni, J Am Coll Cardiol, 2004	13	Agreement between bright-blood MRI angiography and coronary X-ray angiography in identifying coronary aneurysms in KD	(106)
	Comarmond, Am J Cardiol, 2014	27	Myocardial ischemia detected by LGE at CMR was > 5 ×greater in patients with TA compared to matched controls	(133)
	Li, J Comput Assist Tomogr, 2011	42	Whole body MRI; wall thickness and post-contrast signal higher in TAK patients with active disease	(134)
	Koenigkam-Santos, J Clin Rheumatol, 2011	28	GCA/polymyalgia rheumatica; CE-MRA identified extracranial involvement with good interobserver agreement	(135)
	Mavrogeni, Inflamm Allergy Drug Targets, 2013	28	CMR in Churg–Strauss syndrome showed cardiac involvement, with worse prognosis in presence of diffuse sub-endocardial fibrosis	(136)

### Color Doppler ultrasound

**Figure 1:**
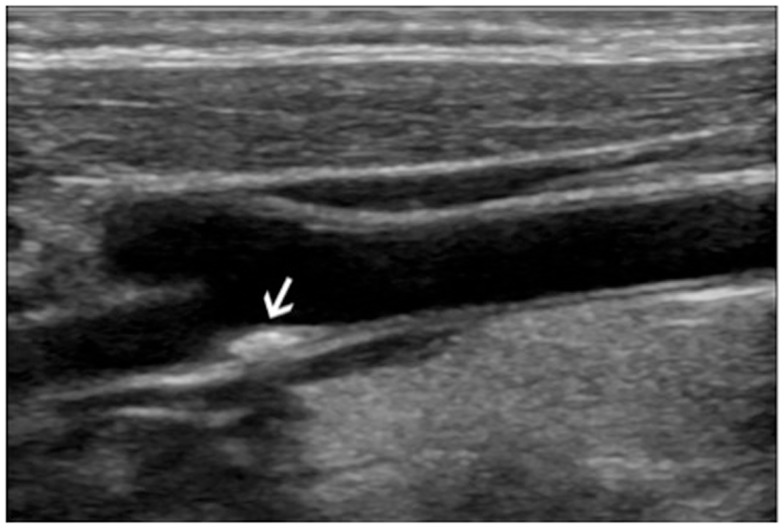
**Ultrasound imaging of the right carotid artery bifurcation of a 44-year-old woman with a 10 years history of systemic lupus erythematosus: the arrows show an atherosclerotic plaque extending toward the internal carotid artery**.

Schmidt et al. pioneered the use of ultrasonography in LVV. They showed that inflamed temporal arteries in GCA were characterized by a dark hypoechoic circumferential wall thickening dubbed the halo sign, which appeared to be edema of the vessel (29). The investigators reported a sensitivity of 73% and a specificity of nearly 100%. Subsequently, temporal artery ultrasonography has increasingly been used to screen patients with suspected GCA. An early meta-analysis showed a sensitivity of 69% and a specificity of 82% for the halo sign using temporal artery biopsy as the reference standard (30). Subsequent meta-analysis showed a sensitivity of 69% and a specificity of 91% (31), and a sensitivity of 69% and a specificity of 89% (32), respectively, when American College of Rheumatology (ACR) criteria were used as reference standard. Interestingly, specificity increased to 100% when presence of the halo sign was bilateral (31). As expected, the quality of the equipment used to perform the examination as well as operator experience was shown to affect diagnostic power of the halo sign (30–32). The halo sign can also be found in inflamed large vessels, which have been shown to be involved in approximately one-third of patients affected by GCA (33, 34). Detection of the halo sign in large vessels further increases ultrasound sensitivity to nearly 100% (35, 36). The equivalent of the halo sign in TAK is called the macaroni sign, a circumferential midechoic thickening of the blood vessel wall (37). Both of these echographic findings have been shown to fade and disappear after initiation of steroid therapy during patients follow-up (38–40). Aside from edema, ultrasound evaluation of the vessel wall may show other vascular alterations. Evaluation of carotid artery wall thickness, i.e., carotid artery intima-media thickness (CIMT), in particular has proved a reliable surrogate measure of atherosclerotic burden and CV risk in the general population (41). CIMT (Figure 1) was shown to be significantly increased in patient affected by a variety of rheumatic diseases: this may represent the result of a multifactorial process in which age and other traditional CV risk factors (i.e., systolic blood pressure, low-density lipoprotein (LDL), cholesterol levels, and body mass index) make a continuous contribution, and interact with inflammation and immunological factors (42, 43). Clear depiction of blood vessel lumen allows detection of stenosis or aneurysmal dilation, which appear as alterations in wall profile as well as flow abnormalities, making ultrasound a valuable tool for monitoring vasculitides complications (3).

### Contrast-enhanced ultrasound

**Figure 2:**
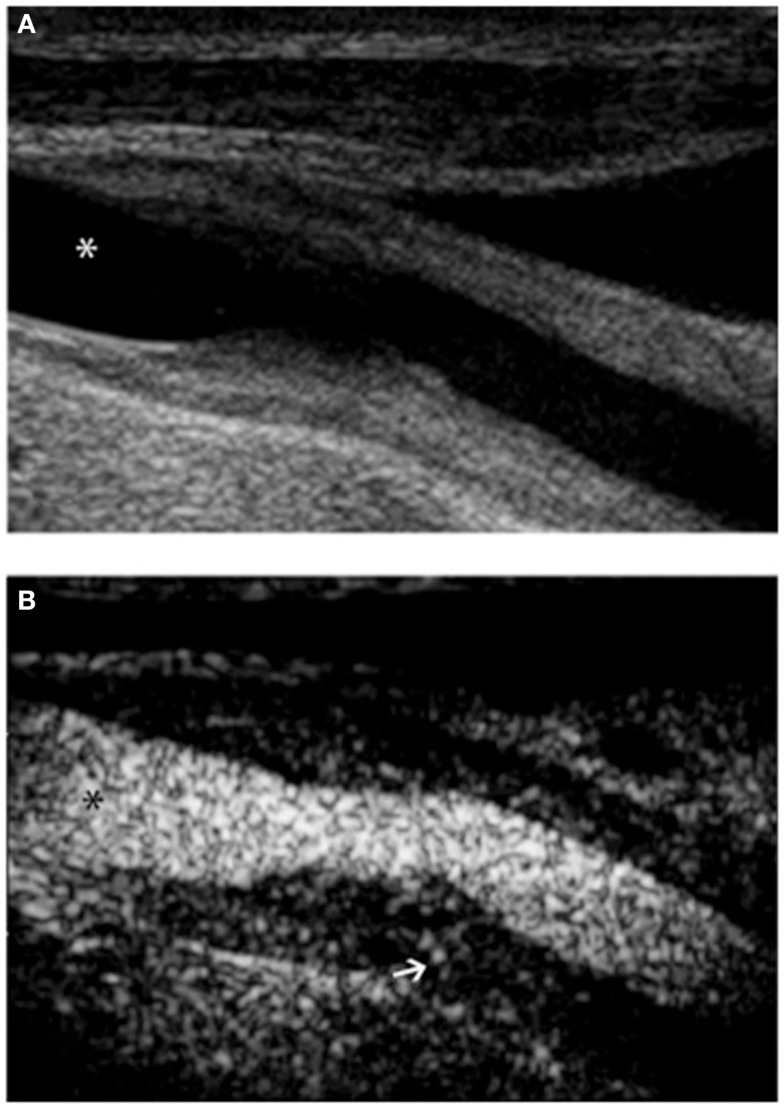
**Ultrasound examination of right proximal common carotid artery of a patient affected by Takayasu arteritis**. B-mode ultrasound **(A)** shows long, smooth concentric thickening of the arterial wall. Contrast-enhanced ultrasound **(B)** using Optison (GE Healthcare, Little Chalfont, UK), a contrast media made up of human albumin microbubbles filled with perflutren, improves definition of the lesion border. Extensive enhancement can be seen within the vessel wall (arrow). In both panels, asterisk marks the vessel lumen.

Several different formulations of ultrasound contrast agents exist. They share the feature of being micro or nano sized, gas-filled particles, known as microbubbles, which remain in the vascular compartment. They generate a hyperechogenic signal because they resonate, i.e., cyclically expand and contract, or release gas when insonated at frequencies used by ultrasound imaging systems (44). When applied to vascular imaging, contrast-enhanced ultrasound (CEUS) is able to enhance the lumen, improving delineation of blood vessel wall (45). In addition, microbubbles allow detection of adventitial neovessels (46), which is a potential marker for atherosclerotic plaque instability (47). Recently, CEUS has been proposed as a potentially useful imaging modality in assessing disease activity in LVV. In early reports by Giordana et al. (48) and Magnoni et al. (49), carotid CEUS was used to diagnose TAK and monitor response to treatment. The authors initially observed circumferential wall thickening in the common carotid artery with multiple vasa vasorum. After treatment, carotid CEUS was repeated and showed progressive reduction in vessel wall and vasa vasorum enhancement, suggesting decrease of inflammatory activity in the carotid artery. CEUS improved definition of borders of the vascular lesion and demonstrated the presence of large amount of contrast, visualized by moving bright spots, on the adventitial side of vascular lesions (Figures 2A,B). The latter phenomenon was interpreted as a signal coming from neovessels. More recently, Schinkel et al. (50) confirmed these results in a pilot study involving seven patients, of which five were affected by TAK and two by GCA. They showed that CEUS significantly improved image quality as compared to standard color Doppler ultrasound and allowed detection of vascularization in carotid vessel wall. CEUS can be employed for molecular imaging (51, 52); unlike freely circulating microbubbles used for vascular opacification, targeted microbubbles are designed to adhere to the endothelium through specific interactions. The adhesion is then detected as increase in echogenicity, which persists after circulating bubbles have been washed away in the site where molecular target is localized. Examples of targets successfully visualized in preclinical experimental models with molecular targeted CEUS include leukocyte adhesion molecules including ICAM-1, VCAM-1, and P-selectin. In the future, this technology will provide the possibility of directly visualizing pathophysiologic events, including inflammatory changes, occurring in the patient’s blood vessels.

The fact that proliferation of vasa vasorum and intimal neovascularization may play a role in atherosclerosis was clearly shown by postmortem studies by Barger et al. (53), who showed that atherosclerotic segments of coronary arteries present a rich vascular network extending from the adventitia to the intima. Atherosclerotic plaque neovascularization may contribute to the progression of a fibrotic, stable lesion to an unstable lesion at high risk of rupture (47, 54). Visualization of intra-plaque neovascularization may therefore provide a way to identify high risk, vulnerable plaques. Detection of blood vessel wall enhancement by CEUS was shown to correlate well with histological evidence of plaque neovascularization, as defined by presence of CD31 positive cells (46, 55). This technique may thus have a relevant future role in risk assessment of atherosclerotic plaques also in patients with immune-mediated disorders.

### Transesophageal echocardiography

Transesophageal echocardiography (TEE) is a semi-invasive ultrasound imaging technique that allows high-quality evaluation of the heart and the aortic root. The exam is carried out using an ultrasonographic transducer mounted on the tip of a modified steerable gastroscope inserted in the esophagus (56). This technique allows correct identification of eventual thoracic aorta and coronary arteries involvement in LVV. Although several reports of utilization of TEE in the evaluation of LVV, especially in the setting of perioperative evaluation (57–59), few clinical studies have been performed using this technique. Bezerra Lira-Filho and colleagues described the most common lesions in 14 patients affected by TAK compared with age-matched controls: aortas of the patients were found to be thicker, more dilated, and stiffer as compared with controls (60). In another study, Espinola-Zavaleta et al. assessed coronary reserve in 15 patients with TAK using contrast-enhanced TEE: 33% of the patients were found to have reduced coronary reserve, while aortic and mitral valve regurgitation was found in 67% and 60%, respectively (61). CEUS can also improve the TEE (62).

## CT and CT Angiography

**Figure 3:**
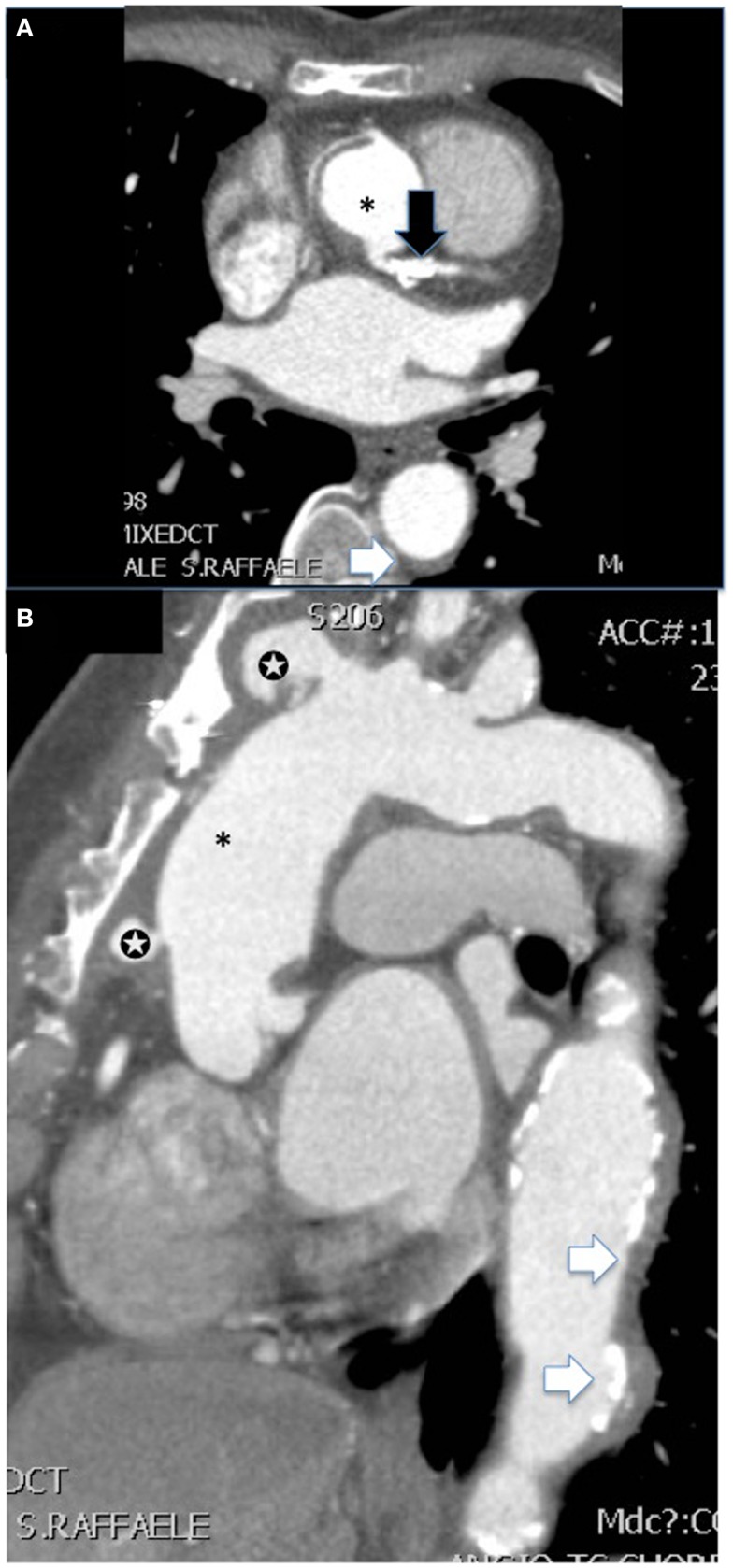
CT angiography [(A) shows axial and (B) sagittal view] of the aorta of a female patient with advanced Takayasu arteritis who underwent previous surgical repair of an ascending aorta aneurysm (*). Several features of the disease are summarized in these images: post-surgical complications such as pseudoaneurysms at the level of proximal and distal anastomosis of the vascular graft with the native aorta (white stars), extensive concentric mural thickening with calcifications of the aorta (white arrows), and presence of a stent in the main steam with a calcific stenosis (black arrow).

Computed tomography is well suited to demonstrate pathological changes in large, deep blood vessels. While it is a widely available and reproducible technique, it involves the use of ionizing radiation and carries a risk connected to the use of iodinated contrast material although radiation dose to the patient has declined steadily in the past few years due to different technological advances (63). CT angiography is able to show alterations both in the wall and in the lumen of affected vessel in LVV (64). In early vasculitis, concentric mural thickening of the involved arteries is typically observed (65, 66). On pre-contrast scanning, the mural thickening has a higher attenuation as compared to the lumen, while in enhanced images it displays a double ring enhancement pattern, most evident in the venous phase (67). In particular, the wall shows a poorly enhanced inner ring and a more obviously enhanced outer ring; it has been proposed that the inner ring represents a swollen intima while the outer one represents active inflammation in the intima and in the media (67, 68). Mural enhancement usually resolves after successful treatment, although its improvement may lag behind clinical and laboratory improvement (69, 70). In advanced disease, CT angiography shows typical late stage complications such as aneurysms, vessel stenosis, or occlusion (66) (Figure 3). Compared with conventional angiography, CT was able to accurately assess stenotic lesions in brachiocephalic trunks, carotid arteries, and subclavian arteries in patients with TAK, with a sensitivity and specificity of 93% and 98%, respectively (71). In addition, coronary arteries assessment with CT was able to show coronary artery lesions in 53% of 111 TAK patients, while only 29% had cardiac symptoms (72). The most common lesions were ostial stenosis, non-ostial stenosis, and coronary aneurysms. In another study, CT was able to detect large-vessel involvement in 27 of 40 patients affected by GCA (73).

## PET and PET/CT

**Figure 4:**
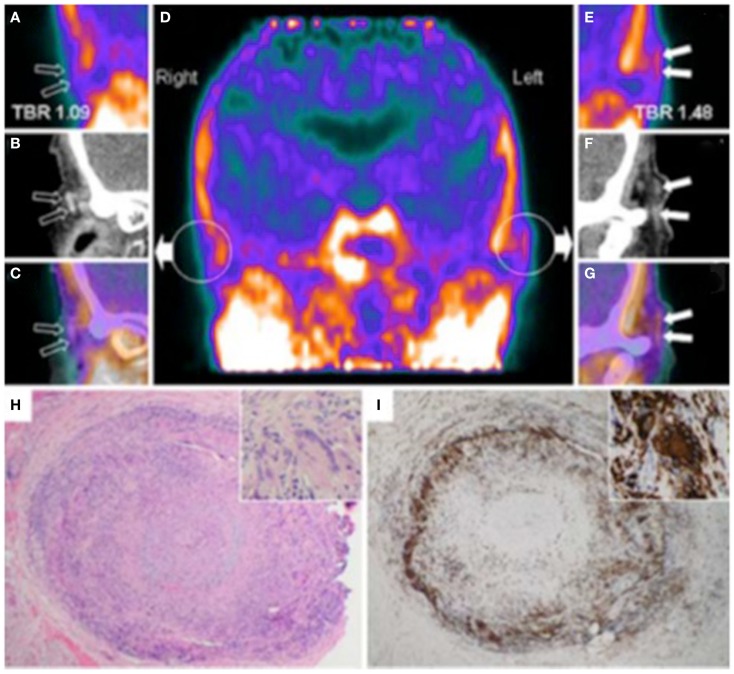
**Hybrid PET with PK11195 and CT angiography imaging of an 88-year-old woman presenting with left scalp tenderness, jaw claudication, and night sweats**. The coronal reconstruction **(D)** and magnifications of PET **(A,E)**, and contrast-enhanced CT **(B,F)** and PET/CT fusion images **(C,G)** show focal PK11195 uptake in the left temporal artery above the zygomatic process (solid arrows) compared to the contralateral artery (open arrows). The arterial target-to-background ratio (TBR) was higher in the left than in the right temporal artery. On CT angiography, the left temporal artery lumen (1.8 mm diameter) was irregular with reduced contrast opacification compared with the contralateral (2.2 mm diameter). The H&E biopsy specimen **(H)** of the left temporal artery (4 × objective) shows transmural granulomatous infiltration (containing activated lymphocytes, macrophages, and multinucleated giant cells), secondary myofibroblast proliferation, and significant intimal thickening leading to luminal obliteration. Large part of the media and the internal elastic lamina are destroyed. The inset (H&E, × 100 objective) demonstrates a multinucleated giant cell. CD68 (macrophage marker, shown in panel I) staining brown with immunoperoxidase (×4 objective) shows dense macrophage infiltration with multinucleated cells (inset, ×100 objective). [adapted with permission from Springer]

Imaging with PET offers unrivaled sensitivity and specificity for research into tissue perfusion, biochemical pathways, and pharmacological mechanisms *in vivo*. The success of PET is founded on the properties of positron emitters. Their short physical half-lives make it possible to administer a tracer dose high enough to obtain useful data, but such that the radiation burden to the patient is acceptably low. A tracer is a measurable substance used to mimic, follow or trace a chemical compound or process without disturbing the process under study. In the case of PET, this is made possible by: (1) the high sensitivity of PET imaging which enables the measurement of radiolabeled tracers administered in picomolar concentrations, which are sufficiently low so as not to disturb the processes under study; and (2) the ability of current PET scanners to perform rapid dynamic imaging and/or list mode acquisitions that provide good temporal resolution. Although it shows great sensitivity to even small amounts of probe mass, PET has a relatively poor spatial resolution (23), compared to CT and MRI. For this reason, coregistration of PET images with contrasted CT images has been developed, to provide good anatomical localization of functional data (74). Using this technique, Gaemperli et al. first reported detection of temporal artery inflammation using PET in a patient with GCA (Figure 4) (75).

### PET and PET CT using FDG

**Figure 5:**
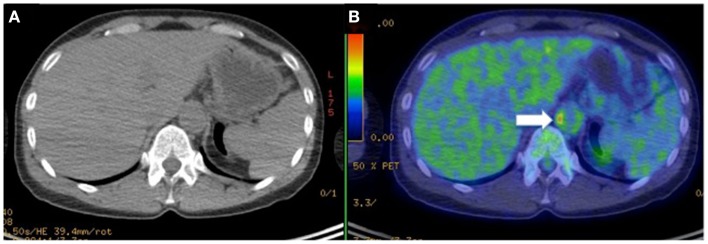
**Hybrid CT (A) with PET imaging (B) to identifies [^18^F]-fluorodeoxyglucose (FDG) accumulation in the vascular wall of abdominal aorta (arrow) in a female patient with Takayasu arteritis**.

[18F]-fluorodeoxyglucose (FDG) is a radiolabeled glucose analog, which competes with glucose for transport across the sarcolemma and phosphorylation by hexokinase and is avidly accumulated by metabolically active cells. Currently, PET imaging with FDG plays a major role in the management of cancer patients (76). Activated inflammatory cells also overexpress glucose transporters and extract increased amounts of glucose and hence FDG (77). In large-vessel vasculitides, FDG uptake in the vascular wall is increased. The uptake is commonly classified on a four-point visual grading system: no uptake (grade 0), less than liver uptake (grade 1), uptake similar to that of the liver (grade 2), and uptake higher than the liver (grade 3) (78). Grades 2 and 3 appear to be specific for vasculitis (79). Alternatively, the semi-quantitative aorta to liver ratio proposed by Hautzel et al. provides an excellent global performance for the diagnosis of GCA, with a sensitivity of 89% and a specificity of 96%, together as robustness as an observer-independent method (80). According to a recent meta-analysis, FDG-PET has a sensitivity of 80% and a specificity of 89% in the diagnosis of GCA, using ACR criteria as reference standard (81). FDG-PET was also shown to be useful in early diagnosis of TAK, showing a sensitivity ranging from 65 to 100% and high specificity (82, 83). Combination of PET with CT imaging was shown to increase diagnostic yield for LVV, allowing for better visualization of tracer accumulation in the vascular wall (84, 85) (see Figure 5 as an example of female patients with inflammation localized at the abdominal aortic wall identified by accumulation of FDG) and visualization of small diameter arteries such as temporal artery (86). Diagnostic accuracy of FDG-PET dramatically decreases after initiation of appropriate therapy (87), providing a valuable tool for disease monitoring (88, 89). Furthermore, PET was shown to reliably predict GCA complications, e.g., aortic dilation (90), but it was unable to identify patient at higher risk of relapse (88). A recent study formally assessed the impact of FDG-PET on the diagnosis of LVV (87). A panel of international experts was asked to diagnose and manage patients with suspect LVV either with or without having access to PET results: PET was shown to significantly increase diagnostic accuracy from 54 to 71%.

### PET/CT using [^11^C]-PK11195

[*N*-methyl-^11^C]-(R)-1-(2-chlorophenyl)-*N*-(1-methylpropil)-3- isoquinoline carboxamide, also known as [^11^C]-PK11195, is a radiolabeled ligand that specifically binds to the translocator protein (TSPO, 18 kDa), formerly known as peripheral benzodiazepine receptor (91). TSPO has been shown to be present at high density in circulating human phagocyte populations, in particular in monocytes and neutrophils (92). Stimulated human monocytes further increase TSPO expression, indicating that its overexpression associates with activation of phagocytes (93). Over the last two decades, [^11^C]-PK11195 has been extensively used in a number of neuroinflammatory and neurodegenerative conditions due to its high uptake in activated microglia and low uptake in neurons (94). Subsequent reports have demonstrated [^11^C]-PK11195 uptake in synovial macrophages of patients affected by rheumatoid arthritis (95). Pugliese et al. first applied [^11^C]-PK11195 in LVV imaging in a small, proof-of-principle study involving 15 patients, predominantly affected by TAK and GCA (96). In this study, all five patients with clinically active vasculitis had markedly increased vascular uptake of the tracer as compared with patients with quiescent disease. Standardized uptake values for [^11^C]-PK11195 correlated well with quantitative total intra-plaque volumes of distribution, calculated from dynamic tissue kinetic modeling using a one-tissue compartment model (97). In one patient, PET/CT images were obtained after a 20-week course of oral corticosteroids: vascular uptake of [^11^C]-PK11195 was markedly reduced, and the reduction was paralleled by clinical improvement and decrease in biochemical markers of inflammation (96). The estimated effective dose for CT was 6.0 ± 0.5 mSv and 2.1 ± 0.2 mSv for PET scan and the mean total effective dose was 8.1 ± 0.6 mSv (96). These values are comparable to those of a cardiac FDG scan (98). Finally, [^11^C]-PK11195 uptake was found to correlate with presence of inflammatory cells in atherosclerotic plaque specimens obtained by carotid artery endarterectomy (99). In the same study, carotid uptake of the radiotracer was higher in patients who had suffered from ischemic stroke, suggesting that this technique may be useful in detecting unstable high-risk plaque (24, 99). Despite these promising results, widespread use of [^11^C]-PK11195 may be limited by its short physical half-life (20 min), which mandates an on-site cyclotron facility. New ^18^F-labeled TSPO ligands, now undergoing preclinical investigation, may overcome some of these barriers (100).

## Magnetic Resonance Imaging

Magnetic resonance imaging is increasingly recognized as a valuable tool in the work-up of patients affected by large- and medium-sized arteries vasculitis (101). MRI has the advantage of avoiding radiations and nephrotoxic contrast medium, while allowing a high-resolution characterization of both vessel wall and lumen. Moreover, systemic vasculitis is increasingly being linked to the occurrence of heart failure secondary to myocardial damage and cardiac magnetic resonance (CMR) has proven to be a refined diagnostic procedure to evaluate both cardiac function and myocardial tissue characterization (102). Avoidance of radiations and tissue characterization capability render MRI an ideal tool not only in defining diagnosis and disease extension but also in evaluating response to treatment and in follow-up. One of the strengths of MRI is the capability of tissue characterization even in basal conditions, before the administration of contrast, so a typical scan includes a pre- and a post-contrast phase; in patients in whom a cardiac examination is also performed, these two steps pertain also to heart evaluation.

### Pre-contrast MRI techniques

**Figure 6:**
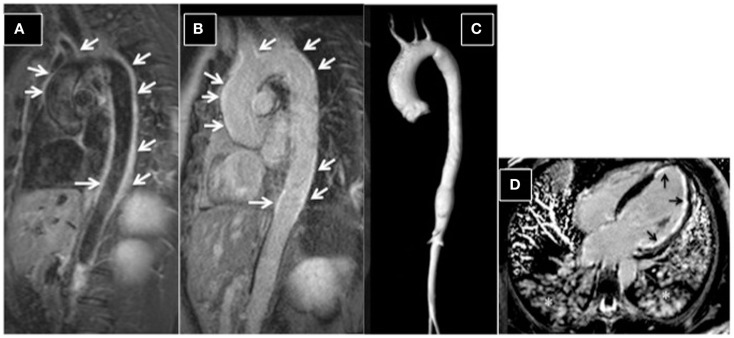
**Aortitis in Behcet’s disease**. **(A)** STIR T2 dark-blood imaging showing extensive edema of the aortic wall (arrows). Post-contrast late-enhancement imaging **(B)** shows extensive enhancement of the aortic wall (arrows). A typical Takayasu aortitis presenting with cardiac arrest and acute myocardial infarction in a 16-year-old girl. **(C)** 3D-MRA reconstructed image, showing dilation of the ascending aorta and narrowing of the descending aorta. **(D)** Post-contrast four-chamber view, showing extensive sub-endocardial and focally nearly transmural myocardial late enhancement (ischemic late-enhancement pattern) of the apical and lateral left ventricular walls. Asterisks highlight the presence of pulmonary edema; the patient was intubated during the scan.

1. Dark-blood morphologic images (HASTE; T1 or T2 turbo spin-echo images), acquired in the axial, sagittal, and coronal planes); in these images blood appears black, while the vessel wall is clearly depicted and its characteristics, such as thickness and regularity can be easily ascertained. Short-tau inversion-recovery images (STIR images) are dark-blood T2-weighted images in which the signal of fat is saturated and that allow the identification of tissue edema (103). This sequence is particularly useful to identify vessel wall active inflammation with edema in the acute phase and to monitor response to therapy and disease activity during follow-up (Figure 6A).
2. Bright-blood morphologic images (single-shot true-FISP images) in which blood appears bright and that allow the evaluation of vessel wall characteristics.
3. Non-contrast angiography. Time of flight angiography (TOF) is the most frequently non-contrast angiography used, especially to study intracranial and peripheral vessels. This technique can be exploited in patients in whom contrast administration is contraindicated (104). The presence of slow flow or vessel tortuosity can impair image quality and represent a limitation of this technique. Bright-blood whole-heart, self-navigated MRI allowing free-breathing is a technique steadily being improved to acquire 3D images of the aorta and coronary arteries without the need of contrast (104, 105). Techniques allowing the evaluation of coronary arteries are particularly useful when these vessels are involved. Mavrogeni et al. demonstrated complete agreement between bright-blood MRI angiography and coronary X-ray angiography in identifying coronary aneurysms in patients affected by KD (106). The avoidance of X-ray exposure is particularly advantageous in this young population and renders MRI the ideal tool for follow-up. Moreover, adding a complete cardiac evaluation with CMR allows a comprehensive evaluation of both the coronary tree and heart muscle (107).

### Post-contrast MRI techniques

Three-dimensional (3D) contrast-enhanced magnetic resonance angiography (CE-MRA) is the corner stone for vessel lumen evaluation with high-spatial resolution (Figure 6C). At higher strength fields (3 T), an improvement in spatial resolution can be obtained with lower contrast dosage (104). Post-contrast acquisitions include T1-weighted images (e.g., VIBE) targeted at the evaluation of vessel wall, allowing the identification of thickening and thrombus stratification and late-enhancement images, obtained with the inversion-recovery technique, showing contrast accumulation (the so-called “late enhancement”) in pathologic vessel walls (Figure 6B).

### Cardiac evaluation with CMR

Systemic vasculitidies can affect the heart either directly, through vasculitis-related myocarditis or indirectly, through myocardial ischemia secondary to coronary artery involvement (102, 108). CMR allows the evaluation of cardiac volumes and function through cine images, the assessment of valvular function with phase-contrast images and the detection of myocardial edema, secondary to inflammation or ischemia through STIR T2-images. Post-contrast images obtained with the inversion-recovery technique highlight pathologic areas of contrast accumulation in the myocardium, identifying scar (Figure 6D). CMR is the ideal tool also to follow-up patients with cardiac involvement, to monitor response to therapy and disease activity.

## Imaging and Biomarkers

The measurement of acute phase reactant, i.e., C-reactive protein (CRP) and erythrocyte sedimentation rate (ESR), is currently recommended for LVV patients follow-up (12) and undoubtedly provides a useful aid for many patients. However, it is established that vascular inflammation and disease progression can occur in the face of normal levels of CRP or ESR, or both (109, 110). In addition, critical analysis of the utility of CRP and ESR, alongside other proposed biomarkers of disease activity in TAK, including soluble adhesion molecules and von Willeband factor, showed that they do not reliably identify active disease (111). The need for more effective markers has prompted the evaluation of several different new molecules, among which Pentraxin 3 (PTX3) deserves a special mention. PTX3 is a member of the pentraxin’s superfamily and is induced by different cell types, including endothelial cells, smooth muscle cells, and mesangial cells upon inflammatory stimulation (112). Dagna et al. first reported that plasma levels of PTX3 were higher in patients with active TAK as compared with patients with inactive disease, and that TAK patients had higher PTX3 plasma concentration than healthy controls and subjects suffering from acute infections (113). Subsequent reports showed high sensitivity and specificity, 82 and 77%, respectively, of PTX3 for the detection of active disease (114, 115), suggesting that it may be of help in early detection of disease recurrence. Monitoring of LVVs by means of biomarkers, however, currently appears to be suboptimal and not fully reliable.

To the best of our knowledge, no systematic study has been performed to assess a correlation between serum level of biomarkers and imaging findings of inflammation. However, many studies report a lack of association between serum levels of CRP and ESR and imaging evidence of active inflammation during follow-up. In particular, a general trend toward persistence of low-grade positive imaging after normalization of acute phase reactants after initiation of treatment is shown for ultrasound (34, 116), FDG-PET (88) and MRI (117). Furthermore, a study performed with PK11195-PET showed that this imaging modality was more effective in identifying clinically active LVV than evaluation of ESR and CRP (96). While none of these findings appears to be conclusive, it is arguable that imaging may be more effective than currently available routine serological biomarkers in the assessment of LVV activity, and may provide an effective means of identifying subclinical active disease.

## Conclusive Clinical Remarks

**Figure 7:**
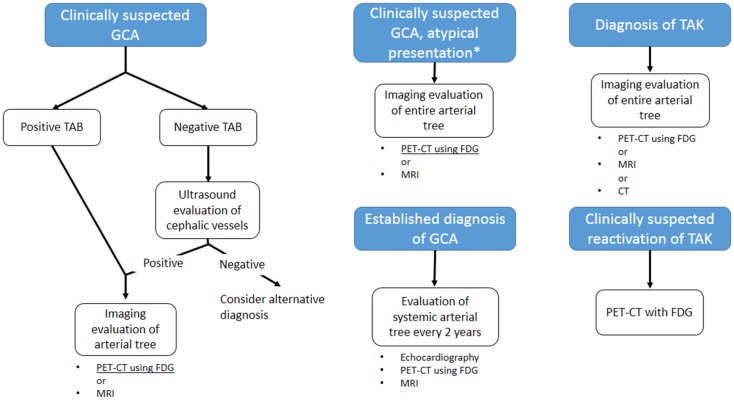
**Proposed guidelines-based algorithm for imaging evaluation of patients with large-vessel vasculitis**. GCA: giant cell arteritis; TAK: Takayasu arteritis; TAB: temporal artery biopsy. *Atypical presentations for GCA include main large-vessel involvement or fever of unknown origin. MRI is a suitable approach only for the former presentation. Currently, no “gold standard” imaging is defined for TAK.

Clinical evaluation still plays a major role in the diagnosis of LVV, also considering the fact that classification criteria have not yet been revised in response to the increased sensitivity of non-invasive imaging techniques (5). The likelihood of early diagnosis, which would be desirable in order to prevent irreversible vascular damage, is not facilitated by the protean manifestation of the diseases, and by the lack of constitutional symptoms in up to 50% of patients (118, 119). The index of suspicion should be held high for patients with unexplained acute phase response, hypertension, or symptoms of ischemia, and should trigger the request for non-invasive imaging (2, 5, 12). Although an early report questioned its utility in the evaluation of LVV (120), imaging of superficial vessels by color Doppler ultrasound was subsequently shown to have a good sensitivity and specificity for the diagnosis of these diseases (31, 32). Changes in ultrasonographic findings have been reported after initiation of an effective therapy (38–40, 121): alongside low economical and biological cost, this makes color Doppler ultrasound a suitable technique for patients’ follow-up. CEUS may further enhance diagnostic power and role in the follow-up of the disease, but evidence is still lacking to warrant its use in clinical practice. For those patients in whom ultrasound examination is negative, PET scanning with FDG is the logical second-line approach. This technique has been shown to greatly increase diagnostic accuracy for LVV (87), and may be particularly useful for those patients with atypical presentation, in which LVV is only one of the several possibilities (122, 123). Although FDG uptake decreases with the initiation of an effective therapy, it is not unusual to detect persistent low-grade uptake in the involved arteries of patients who have normal acute phase reactants (124), which may be due to the persistent inflammatory infiltrates that can be detected in surgical specimens from patients with quiescent disease (110). This fact currently limits the utility of PET in patients’ follow-up. These shortcomings have led investigators to explore new ligands for detection of vascular inflammation, such as [^11^C]-PK11195, which has not been extensively validated yet, but for which promising preliminary result exist (96). MRI imaging can represent an alternative second-line examination, allowing complete evaluation of the aorta and its branches, but appropriate visualization of blood vessel lumen and wall necessitates of dedicated protocols not widely available (125, 126). In addition, MRI allows thorough evaluation of the heart, which is frequently involved, albeit sometimes silently, in LVV. The lack of ionizing radiation makes it suitable for repetition in patients follow-up. CT imaging has similar indications to MRI for what concerns blood vessels examination, and is more widely available. It may have a role in the evaluation of coronary arteries involvement in these patients. However, it involves the use of ionizing radiations and nephrotoxic iodinated contrast, which questions its safety for repeated exams in the follow-up of the patients. Current guidelines, while supporting the use of non-invasive imaging in the diagnosis and management of LVV (12), do not give systematic indications its use, deferring the selection of the approach to the practicing physician preferences and local expertize. The use of ultrasonography is well established in the diagnosis of GCA: while a positive ultrasound finding does not replace temporal artery biopsy for the diagnosis, it may provide the definite diagnosis for biopsy negative patients with a strong clinical suspicion for cephalic vessel GCA (127). Ultrasound may thus be the first imaging approach in such patients. FDG-PET, especially if combined with anatomical CT imaging, appears to be particularly appropriate subsequently, as it allows to detect lesions throughout the entire arterial tree (128). PET imaging may be particularly appropriate for patients affected by GCA (127) in whom large-vessel involvement and systemic symptoms, e.g., limb claudication, predominate, and in patients with atypical presentation, i.e., fever of unknown origin (122). MRI may have similar indications, although less established. In the subsequent management of the patient, imaging of the arterial tree is recommended every 2 years in order to monitor for lesion progression: echocardiogram for early detection of proximal aortic aneurysm, PET or MRI may be included (127, 129). For what concerns TAK, guideline indications are even less precise. A thorough assessment of the arterial tree is currently recommended upon diagnosis, and MRI, PET, and CT are well suited for this purpose (128), while none of these techniques appear to provide a “gold standard” (130). The use of ultrasound is not well established in this disease (131), but it may provide the tool for non-invasive assessment of cephalic vessel, especially carotid artery. The detection of the “halo sign” may provide information on disease activity. One of the major challenges in therapeutic management of TAK is in fact recurrence: even with effective corticosteroid therapy, up to 72% of the patients experience multiple recurrences within 6 months (119). While no routine imaging follow-up is currently recommended for early detection of recurrence, FDG-PET may be the imaging technique of choice upon clinical suspicion due to its strong correlation with disease activity (132). Diagrams in Figure 7 summarize the suggested imaging approach.

## Conflict of Interest Statement

The authors declare that the research was conducted in the absence of any commercial or financial relationships that could be construed as a potential conflict of interest.
